# Vitamin D Supplementation and its Effect on Relapse Frequency and MRI Activity in Adults With Multiple Sclerosis: A Systematic Review

**DOI:** 10.1002/brb3.71692

**Published:** 2026-08-18

**Authors:** Kamren Hall, Jessica Renteria, Elizabeth Fernandez, Janique Abrahams, Saajan Bhakta

**Affiliations:** ^1^ Kansas College of Osteopathic Medicine Wichita Kansas USA

**Keywords:** MRI, multiple sclerosis, relapse rate, vitamin D

## Abstract

**Introduction:**

Literature evidence links vitamin D supplementation to clinical outcomes in multiple sclerosis (MS), yet no systematic review has focused specifically on relapse rate and MRI activity. Thus, this systematic review aimed to evaluate the effect of vitamin D supplementation on relapse rate and magnetic resonance imaging (MRI) disease activity in adults with clinically diagnosed MS.

**Methods:**

This systematic review was conducted in accordance with the Preferred Reporting Items for Systematic Reviews and Meta‐Analysis (PRISMA) guidelines. PubMed, Cochrane, EBSCO, and Web of Science were searched for studies discussing vitamin D supplementation in adults with diagnosed MS. Annualized relapse rate (ARR) and changes in MRI activity were the primary outcome measures. Non‐randomized studies and randomized controlled trials were assessed for their risk of bias using the Cochrane ROBINS‐I and RoB‐2 tools, respectively.

**Results:**

Of 304 records identified, 127 duplicates were removed and 177 were screened, with 11 primary studies (14 reports) included after full‐text review. Across studies, vitamin D supplementation did not suggest a reduced ARR with most randomized trials reporting no significant difference between groups. One trial using alfacalcidol provides evidence for a reduction in relapse risk, but this was not replicated. MRI outcomes were more variable, with several studies showing reductions in inflammatory lesion activity, while others found no significant effect. Overall, results were inconsistent, and due to heterogeneity in study design and reporting, findings were synthesized narratively without meta‐analysis.

**Conclusion:**

Vitamin D supplementation does not consistently reduce relapse rates in adults with MS, although some studies report improvement in MRI activity. The overall certainty of evidence is limited by variation in study design, patient population, cohort size, and risk of bias. Current data does not support vitamin D as a disease‐modifying therapy beyond correction of deficiency; further, larger, standardized trials are needed to clarify its clinical role.

## Graphical abstract

This systematic review examined whether vitamin D supplementation reduces disease activity in adults with multiple sclerosis. Across 11 primary studies, vitamin D was not consistently associated with reduced relapse frequency, though MRI‐based measures showed a modest and inconsistent signal, leaving its clinical benefit uncertain.

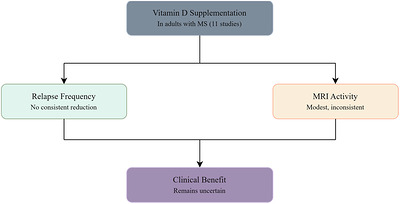

**Abbreviations**
ARR: annualized/annual relapse rate
DMT: disease‐modifying therapy
GELs: gadolinium‐enhancing lesions
MeSH: medical subject headings
MRI: magnetic resonance imaging
MS: multiple sclerosis
PRISMA: Preferred Reporting Items for Systematic Reviews and Meta‐Analyses
RRMS: relapsing‐remitting multiple sclerosis
UVR: ultraviolet radiation

## 1 Introduction

Multiple sclerosis (MS) is a chronic, immune‐mediated demyelinating disease of the central nervous system. The main types of MS include relapsing‐remitting multiple sclerosis (RRMS), secondary progressive MS, and primary progressive MS. The most common type of MS is RRMS (Galus et al. 2025). More than 2 million individuals are affected worldwide, most of whom are young adults (Zheng et al. 2018). Many correlations have been studied regarding factors that may cause or worsen MS. A recently studied topic has been vitamin D supplementation and how it can affect the disease course of MS.

Vitamin D is a fat‐soluble vitamin that is present in some foods and is endogenously synthesized in the skin (Jagannath et al. 2018). The metabolism of vitamin D begins in the liver, where it is converted to calcidiol (25OHD). Next, the kidneys convert it to calcitriol (1,25‐dihydroxyvitamin D) (Jagannath et al. 2018). Vitamin D stimulates anti‐inflammatory pathways that are protective in autoimmune diseases such as MS (Galus et al. 2025). Activation of vitamin D receptors has been shown to inhibit B‐ and T‐lymphocyte proliferation and reduce the production of inflammatory cytokines seen in MS (Mosayebi et al. 2011). Additionally, vitamin D suppresses activation of microglia and astrocytes and promotes oligodendrocyte proliferation, suggesting potential neuroprotective and remyelinating effects. It is associated with anti‐inflammatory cytokines, including transforming growth factor‐β, interleukin‐10, and interferon γ. There is comparably less effect on pro‐inflammatory cytokines such as interleukin‐17 (Mosayebi et al. 2011; Galus et al. 2023). In association with the anti‐inflammatory benefits of vitamin D supplementation, individuals with MS have shown improvement in relapse frequency. Studies have shown up to 12% decrease in relapse rate for every 10 nmol/L of 25‐hydroxy vitamin D (Kampman et al. 2012).

Given this relationship, low vitamin D levels correlate with a higher risk of developing MS, as well as experiencing a more severe disease course. For example, vitamin D levels are influenced by direct ultraviolet radiation (UVR) exposure. The higher the latitude, the lower the UVR exposure, and consequently, less vitamin D and a higher correlation with MS (Zheng et al. 2018). Conversely, higher serum vitamin D levels have been associated with reduced disease activity and improved clinical outcomes (Galus et al. 2023). Possibly related to this link, trials have been conducted to evaluate the vitamin D supplement in patients with MS (Mahler et al. 2024).

Magnetic resonance imaging (MRI) scans also play an essential role in the diagnosis of MS. Scans detect demyelinating lesions that indicate the progression of the disease (Galus et al. 2023). On MRI, demyelinating lesions typically appear hyperintense on T2‐weighted. When lesions are active, associated inflammation is linked to gadolinium enhancement, indicating ongoing disease activity. Studies have shown statistically significant associations between vitamin D and reduced number of new T2‐weighted lesions in addition to gadolinium‐enhancing lesions (GELs) as seen on MRI in MS. Positive radiological outcomes were observed with vitamin D supplementation, including lower brain atrophy (Galus et al. 2023).

The beneficial role of vitamin D in patients with MS remains unsolved, as clinical trial data have not consistently supported the findings suggested by epidemiologic and mechanistic evidence. There are current gaps in knowledge that need to be further evaluated. This review aims to evaluate the effect of vitamin D supplementation on relapse frequency and MRI activity on adults with MS.

## 2 Methods

### 2.1 Design

This study was conducted from October 6, 2025 to March 26, 2026 following the Preferred Reporting Items for Systematic Reviews and Meta‐Analyses (PRISMA) guidelines.

### 2.2 Search Strategy

A search strategy was conducted utilizing the databases PubMed, Cochrane Reviews, Cochrane Trials, EBSCO, and Web of Science. Articles were identified using a combination of Medical Subject Headings (MeSH) and free‐text terms, which included the following core phrases depicted in Table 1. These terms were combined with additional terms to target annualized relapse rate (ARR) and GELs, along with Boolean operators (e.g., AND and OR) in order to optimize the sensitivity of the search. This search strategy was designed to be reproducible. Results of the search inquiry are shown in Table 1.

**TABLE 1:** Search strategy and database yields.

Search strategy	PubMed	Cochrane reviews	Cochrane trials	EBSCO: Medline ultimate	Web of Science (core collection)	Total
(“Multiple Sclerosis”[MeSH] OR “Multiple Sclerosis, Relapsing‐Remitting”[MeSH] OR multiple sclerosis OR MS OR relapsing‐remitting MS OR RRMS)	65,079	5524	55,919	94,731	34,432	255,685
(“Vitamin D”[MeSH] OR “Cholecalciferol”[MeSH] OR “Ergocalciferols”[MeSH] OR “Calcifediol”[MeSH] OR vitamin D OR vitamin D3 OR vitamin D2 OR cholecalciferol OR ergocalciferol OR oral vitamin D OR calcifediol)	16,717	22,437	21,648	14,741	6071	81,614
(“Recurrence” OR “Disease Progression” OR relapse OR attack OR flare OR exacerbation OR “annualized relapse rate” OR ARR OR “Magnetic Resonance Imaging” OR “Gadolinium DTPA” OR MRI OR lesion OR “gadolinium enhancing lesion” OR “contrast enhancing lesion” OR “T1 hypointense lesion” OR “brain atrophy” OR NEDA OR “no evidence of disease activity” OR “new or enlarging T2 lesions”)	394,439	4698	214,639	350,203	185,616	755,550
1 + 2 + 3	130	0	56	100	27	313
1 + 2 + 3 Limit: English	128	0	56	99	27	310
1 + 2 + 3 Limit: English + last 20 years	125	0	56	96	27	304
Duplicates removed (records marked as ineligible by automation tools)						127 Screen: 177

### 2.3 Study Selection

References were downloaded and managed via Rayyan AI. Rayyan AI algorithmically identified duplicates, which were then verified during screening. After duplicates were removed, the selection process included four independent reviewers who screened both title/abstract based on eligibility criteria, and any disagreements were discussed and resolved by consensus. Once the ineligible references were removed, all four reviewers blindly screened the full texts of all remaining papers, and conflicts were discussed to decide which would be included in this systematic review.

### 2.4 Eligibility Criteria

Included in the study were randomized controlled trials, comparative cohort studies, and case‐control studies that followed distinct criteria. All studies considered must have included human patients over the age of 18 with diagnosed MS, used vitamin D as a discrete and discernable intervention (studies may have included multicomponent interventions and therapies), been published within the past 20 years (2005 or later), been published in English language (due to lack of translator/translation resources), and required comparison with a placebo, no intervention, standard care, or a quantifiable exposure category.

Excluded from the study were case reports, narrative reviews, systematic reviews, and meta‐analyses (used only for contextual discussion) and expert opinions (gray literature or letters to an editor) that did not follow the previously mentioned criteria. Studies not considered included animal studies, in vitro studies, or pediatric populations (under 18), studies where vitamin D and its effects are not separable in the case of multicomponent interventions, studies published prior to 20 years ago (older than 2005) or not published in English.

### 2.5 Data Collection and Extraction

Data extraction was independently performed by multiple reviewers using a standardized data extraction form to ensure consistency across studies. Each reviewer extracted data from their assigned studies, and the extracted variables were subsequently compared across reviewers. Any discrepancies were resolved through discussion to reach consensus, automated tools were not utilized in the data collection process, and attempts to contact study investigators for missing or confirmation of data were not required nor performed.

The standardized extraction form captured study characteristics (e.g., authorship, sample size, and intervention details) as well as primary outcomes of interest included measures relevant to disease activity in MS. Specifically, measurements such as ARR, new or enlarged T2‐weighted GELs, changes in total lesion volume, and measures of statistical significance such as confidence intervals and p‐values were included when provided in the reference.

Given variability in reporting across studies, not all possible outcome measures or timepoints reported in each study were extracted; instead, outcomes that were consistently reported and those clinically relevant to this review were prioritized. Additional variables extracted from the studies included general characteristics such as authorship, sample size, intervention details, and secondary effects. If information was missing or unclear, no imputation methods were applied, but what was available was used as reported, and limitations were considered during the interpretation of the results.

### 2.6 Statistical Analysis and Synthesis

The effect measures in the review were reported as presented in the original studies. Continuous outcomes, such as changes in total lesion volume, were summarized using mean differences or changes from baseline, while clinical outcomes, such as ARR, were reported using rate‐based measures. Statistical significance was reported using confidence intervals and *p*‐values when available. No standardized effects were calculated.

Included studies reported outcomes related to MS disease activity, including ARR, new or enhancing T2‐weighted GELs, or changes in total lesion volume. Data were extracted from the studies and reported without transformation, and missing data were not imputed. Results were summarized descriptively using structured tables. Findings were synthesized narratively rather than through meta‐analysis.

### 2.7 Quality and Bias Assessment

To promote a thorough and transparent evaluation of the studies that were included in this analysis, previously established Cochrane risk‐of‐bias frameworks were applied. Randomized controlled studies were assessed using the RoB 2 tool, while non‐randomized studies were evaluated with the ROBINS‐I instrument. Risk of bias assessments for included studies are summarized in Tables 3 and 4. Both tools were utilized in accordance with guidance from the Cochrane Handbook for Systematic Reviews of Interventions. At least two reviewers independently examined each study to assess the methodological quality and to detect potential bias. For randomized trials, risk of bias was classified as low, some concerns, or high, whereas non‐randomized studies were categorized as low, moderate, serious, or critical risk of bias. Any discrepancies between the two reviewers were resolved through discussion until consensus was achieved, ensuring consistency across evaluations of all studies included.

Due to the limited number of studies that were included, a formal statistical assessment of publication bias utilizing a funnel plot was not performed. Instead, reporting bias was evaluated qualitatively by assessing the consistently reported outcomes and study objectives or methods independently. The overall certainty of the evidence was not formally investigated using a standardized framework such as GRADE. Confidence in our findings was assessed qualitatively by considering key factors of the study, including but not limited to the study design, risk of bias, consistency of results across studies, and the precision of reported outcomes. Each of these elements was used to discuss the author's interpretation of the strength and reliability of the evidence that was presented.

## 3 Results

### 3.1 Study Selection

**FIGURE 1:**
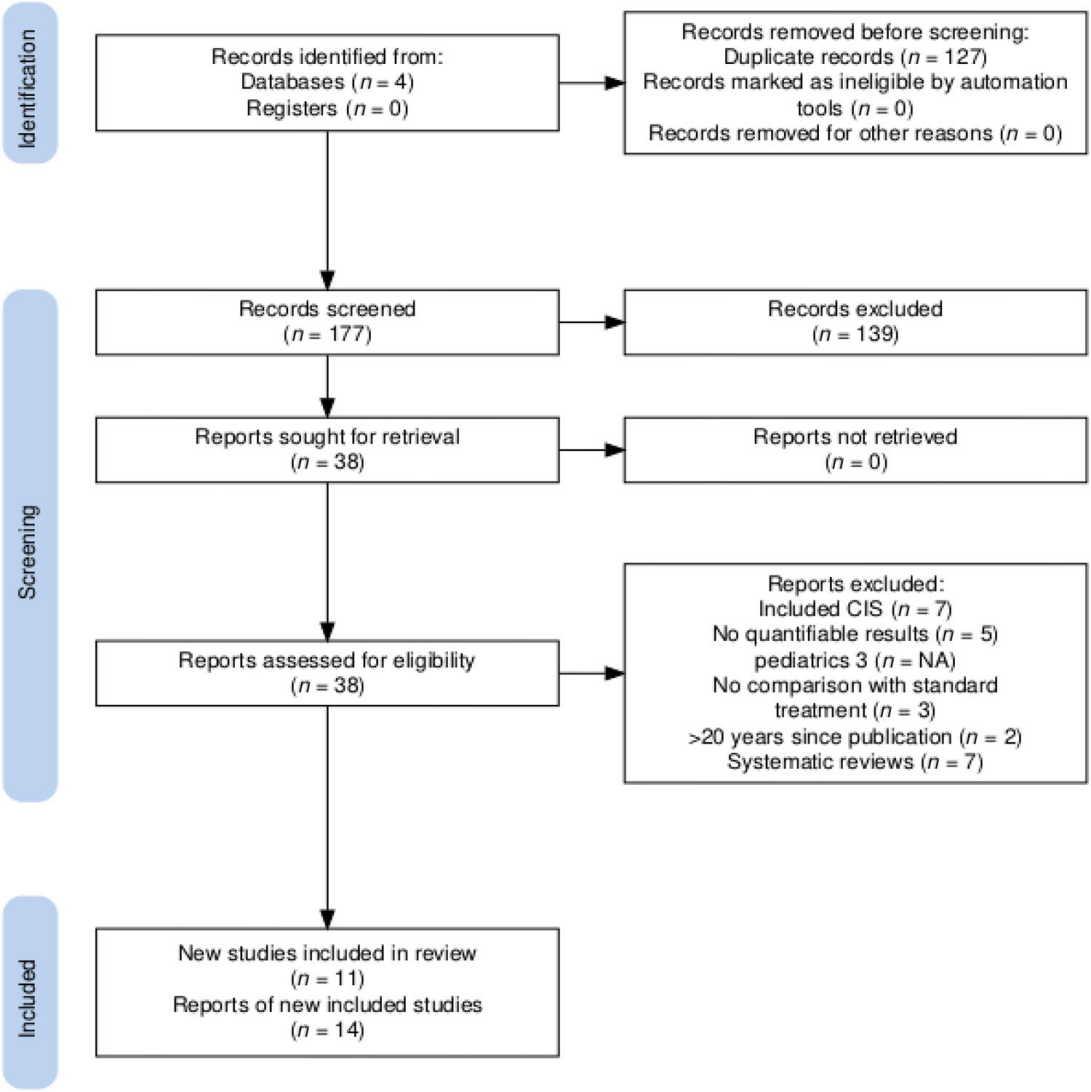
PRISMA 2020 flow diagram.

The initial database search yielded 304 records, from which 127 duplicates were identified and removed using Rayyan AI, which left 177 records to be screened by title and abstract. Of those, 139 were excluded and 38 records were then moved forward for full text screening. Note that 27 of the 38 were excluded for reasons as follows: studies focused on clinically isolated syndrome rather than MS (*n* = 7), studies without quantifiable outcomes (*n* = 5), studies including pediatric age population (*n* = 3), studies without a comparator arm (*n* = 3), studies published over 20 years ago (*n* = 2), and systematic reviews or meta‐analyses, which were excluded from primary evidence synthesis and used only for contextual comparison in the discussion (*n* = 7). That left 11 primary studies included in this review, of which a few studies had more than one associated report, such as a trial registry entry alongside a published paper, yielding a total report of 14 rather than 11. The full breakdown of this process can be seen in Figure 1.

### 3.2 Study Characteristics

The 11 included studies were overall seen to be heterogeneous in terms of design, population, and how vitamin D was being used as an intervention. When primary studies were examined, 8 of 11 were randomized controlled trials, one was a post hoc analysis pulled from two phase III fingolimod trials (Hongell et al. 2018), and two were a prospective observational cohort that followed patients for either 2 or 4 years (Galus et al. 2023, 2025). Most of RCT populations contained RRMS patients, though two of the trials incorporated a broader MS population without restricting to RRMS. Five of the trials included vitamin D using an add‐on design, meaning it was received on top of an already existing disease‐modifying therapy, most commonly a form of interferon β (Camu et al. 2019; Hupperts et al. 2019; Soilu‐Hänninen et al. 2012). Doses used across trials varied, from as low as 1 µg/day of alfacalcidol up to 40,000 IU/day of cholecalciferol, and additionally, follow‐up ranged from 6 months to as long as 48 months (Achiron et al. 2015; Burton et al. 2010). Full characteristics and outcome data for primary data can be found in Table 2.

**TABLE 2:** Characteristics and outcomes of included primary studies: Relapse rate and MRI endpoints.

Study (year)	Design/setting	Population & N	Intervention vs. comparator	Follow‐up	Relapse outcomes	MRI outcomes
Achiron et al. (2015)	RCT, DB, placebo‐controlled	MS; *N* = 158	Alfacalcidol 1 µg vs. placebo	6 months	Relapse benefit reported (OR ∼0.200 [0.090 – 0.530], *p* < 0.001; relapse counts 8/80 vs. 25/78)	MRI not evaluated
Burton et al. (2010)	Phase I/II controlled dose‐escalation	MS; *N* = 49	High‐dose D3 up to 40,000 IU/day vs. control (allowed low‐dose)	52 wks	ARR during trial year 0.260 vs. 0.450, *p* ≈ 0.090 (exploratory)	MRI outcomes NR
Camu (2019)	RCT, DB, placebo‐controlled (add‐on IFNβ‐1a)	RRMS; *N* = 129 start (∼90 completed)	D3 (≈7143 IU/day equivalent) vs. placebo	96 wks	ARR 0.094 vs. 0.110; HR 0.801 (0.403 – 1.454), *p* = 0.43	MRI benefit on select measures (new T1 lesions/T1 hypointense volume); new/enlarging T2 & Gd+ often NS
Kampman et al. (2012)	RCT, DB, placebo‐controlled	RRMS; *N* = 71	D3 20,000 IU/week (+ calcium) vs. placebo (+ calcium)	96 wks	ARR difference 0.100 (−0.070–0.270), *p* = 0.25; on‐study ARR difference 0.060 (−0.080–0.200), *p* = 0.420	MRI outcomes NR
Mosayebi et al. (2011)	RCT	MS; *N* = 62	D3 300,000 IU IM monthly vs. placebo IM	6 months	Relapse/EDSS changes NR/NS	Gd+ lesions: no significant difference reported
Soilu‐Hänninen et al. 2012)	RCT, DB, placebo‐controlled (add‐on IFNβ‐1b)	RRMS; *N* = 66	D3 20,000 IU/week vs. placebo	12 months	ARR 0.260 vs. 0.330, NS	Gd+ lesions 0.100 vs. 0.600; *p* = 0.004. T2 lesion volume trend NS (*p* ≈ 0.105)
Hongell et al. (2018)	Post hoc analysis of RCTs (FREEDOMS/FREEDOMS II)	RRMS; *N* = 829 (Vit D use vs. non‐use)	Daily users vs. non‐users (dose NR)	24 months	ARR: no difference across Vit D groups (NS)	T2 lesion‐free proportion favored daily users (*p* = 0.038 at M12; *p* = 0.009 at M24); PBVC better at M12 (*p* = 0.018), M24 NS
Hupperts (2019)	Phase II RCT, DB, placebo‐controlled (add‐on IFNβ‐1a)	RRMS; *N* = 229 ITT	D3 oil 6,670 IU/day → 14,007 IU/day vs. placebo oil	48 wks	ARR observed 0.280 vs. 0.410; modeled IRR 0.800 (0.510–1.260), *p* = 0.340; time‐to‐first relapse HR 0.740 (0.380–1.430), *p* = 0.370	CUA lesions IRR 0.680 (0.520–0.890), *p* = 0.0045; T2 lesion volume change significant (*p* = 0.035)
Cassard (2023)	Multicenter RCT (add‐on glatiramer acetate)	RRMS; *N* = 172	5000 IU/day vs. 600 IU/day (both + GA)	2 years	Confirmed relapse risk HR 1.170 (0.670 – 2.050), *p* = 0.570; ARR 0.340 vs. 0.200, *p* = 0.070	New/enlarging T2 lesions rate ratio 1.470 (0.880–2.460), p = 0.140; brain volume/GMV changes NS
Galus et al. (2023)	Prospective observational cohort	RRMS; *N* = 133 start → *N* = 92 at 24 months	Vitamin D supplementation (D3, typically 1000–4000 IU/day) vs. no supplementation	24 months	Relapses: NS (0.200 ± 0.500 vs. 0.100 ± 0.400; *p* = 0.698)	New T2 lesions: 0.4 vs. 0.8 (*p* = 0.034); Gd+ lesions NS (*p* = 0.094)
Galus et al. (2025)	Prospective observational cohort	RRMS; *N* = 132 start → 97 supplemented/35 non	D3 supplementation (mean ∼2600 IU/day) vs. no supplement	48 months	Relapse outcomes NR/NS	Inflammatory MRI activity NS; brain atrophy measures showed some significant differences (observational; confounding likely)

*Note*: CUA = combined unique active; DB = double‐blind; EDSS = Expanded Disability Status Scale; GA = glatiramer acetate; Gd+ = gadolinium‐enhancing; HR = hazard ratio; IFNβ = interferon beta; IM = intramuscular; IRR = incidence rate ratio; ITT = intention‐to‐treat; mo = months; NR = not reported; NS = not significant; OR = odds ratio; PBVC = percent brain volume change; RCT = randomized controlled trial; T2 = T2‐weighted; wks = weeks.

### 3.3 Risk of Bias Assessment

Results of risk of bias assessments are presented in Table 3 (non‐randomized studies, ROBINS‐I) and Table 4 (randomized controlled trials, RoB‐2).

**TABLE 3:** Risk of bias assessment for non‐randomized studies using Cochrane ROBINS‐I tool.

Study	D1	D2	D3	D4	D5	D6	D7	Overall
Galus et al. 2025	Serious	Moderate	Moderate	Moderate	Moderate	Low	Moderate	Serious
Hongell et al. 2018	Serious	Serious	Serious	Moderate	Moderate	Low	Serious	Serious
Galus et al. 2023	Serious	Moderate	Moderate	Serious	Moderate	Serious	Serious	Serious

*Note*: The Cochrane ROBINS‐I Tool was used to assess the risk of bias for the non‐randomized studies included in the review across the following domains: Domain 1 (D1)—Risk of bias due to confounding; Domain 2 (D2)—Risk of bias in classification of interventions; Domain 3 (D3)—Risk of bias in selection of participants into the study (or into the analysis); Domain 4 (D4)—Risk of bias due to deviation from intended interventions; Domain 5 (D5)—Risk of bias due to missing data; Domain 6 (D6)—Risk of bias arising from measurement of the outcome; and Domain 7 (D7)—Risk of bias in selection of the reported result.

**TABLE 4:** Risk of bias assessment for randomized controlled trials using the Cochrane RoB‐2 tool.

Study	D1	D2	D3	D4	D5	Overall
Achiron et al. 2015	Low	Low	Low	Some	Some	Some
Mosayebi et al. 2011	High	Low	Some	Low	High	High
Kampman et al. 2012	Low	Some	Low	Low	Low	Some
Camu et al. 2019	Some	Low	High	Low	Some	High
Soilu‐Hänninen et al. 2012	Low	Low	Some	Low	Low	Some
Burton et al. 2010	High	High	Some	High	High	High
Cassard et al. 2023	Low	Low	Low	Low	Low	Low
Hupperts et al. 2019	Low	Some	Some	Low	Some	Some

*Note*: The Cochrane RoB‐2 tool was used to assess the risk of bias for the randomized controlled trials included in the review across the following domains: Domain 1 (D1)—bias arising from the randomization process; Domain 2 (D2)—bias due to deviations from intended intervention; Domain 3 (D3)—bias due to missing outcome data; Domain 4 (D4)—bias in measurement of the outcome; and Domain 5 (D5)—bias in selection of the reported result.

Risk of bias was assessed utilizing the ROBINS‐I tool for the three non‐randomized control studies and the RoB‐2 tool was used for eight randomized control trials included in this study as seen in the tables above. Among the non‐randomized studies, all three were judged to have an overall serious risk of bias. Most of the concerns surrounding these studies included potential confounding factors, improper selection of participants, and selection of the reposted result, as several studies posed a serious risk among those respective domains. Between the randomized control trials, four studies were considered to have some concerns for bias, three had high concerns for bias, and one had a low concern for bias. Overall, the studies included evidence that was characterized in a moderate‐to‐high methodological limitation, which must be considered when interpreting the reported associations between vitamin D supplementation and clinical outcomes in patients diagnosed with MS.

### 3.4 Relapse Outcomes

When examining relapse data reported from included studies, a clear pattern emerged: vitamin D supplementation did not consistently reduce relapse frequency or ARR. Five of the six RCTs reporting relapse data (Burton et al. 2010; Camu et al. 2019; Hupperts et al. 2019; Kampman et al. 2012; Soilu‐Hänninen et al. 2012) showed no statistically significant benefit, and that was consistent regardless of whether the trial tested vitamin D supplementation as a standalone therapy or as an add‐on to an existing disease‐modifying therapy (DMT).

The SOLAR trial was the largest primary RCT included in this review; the trial with 229 participants showed no association between vitamin D supplementation and relapse rate. The observed ARR was lower in the vitamin D supplementation group (0.28 vs. 0.41), but the modeled incidence rate ratio (IRR 0.8 [0.51–1.26], *p* = 0.34) and time to first relapse (HR 0.74 [0.38–1.43], *p* = 0.37) did not hold up (Hupperts et al. 2019; SOLAR 2026). The VIDAMS trial, with 172 participants showed high‐dose vitamin D at 5000 IU/day did not reduce relapse risk compared to low‐dose 600 IU/day, with both groups also having concurrently taken glatiramer acetate (HR 1.17 [0.67–2.05], *p* = 0.57). The ARR in the VIDAMS favored the other direction, 0.34 in the high‐dose group versus 0.20 in the low‐dose group (*p* = 0.07) (Bhargava et al. 2014; Cassard et al. 2023; VIDAMS 2023). The CHOLINE trial followed 129 participants over 96 weeks and showed ARRs of 0.094 versus 0.110 between groups, with a hazard ratio that did not favor vitamin D supplementation (HR 0.801 [0.403–1.454], *p* = 0.43) (Camu et al. 2019). Kampman et al. (2012) with 71 participants found ARR differences at both timepoints assessed (*p* > 0.05), and Soilu‐Hänninen et al. (2012) with 66 participants, saw a lower ARR with vitamin D supplementation (0.26 vs. 0.33). Burton et al. (2010) utilized a dose‐escalation trial with 49 participants and found an exploratory ARR of 0.26 versus 0.45 (*p* ≈ 0.09). Across these trials, ARR differences were not statistically significant.

**FIGURE 2:**
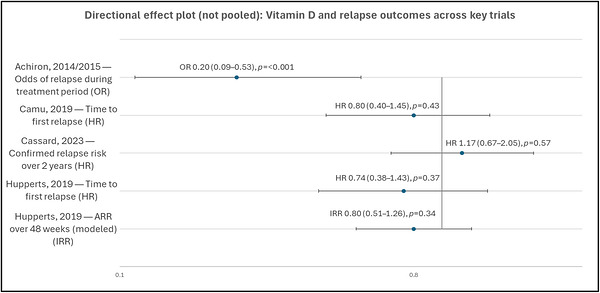
Directional effect plot (not pooled): Vitamin D and relapse outcomes across key trials. Effect estimates (ARR ratio/relapse risk/time‐to‐first relapse) are shown with 95% CIs. Values < 1 favor vitamin D; the vertical line at 1 indicates no effect. Effect measures vary by study and are not directly comparable across trials.

Achiron et al. was the one trial that did show relapse benefit, where participants received 1 µg/day of alfacalcidol. Participants reduced the odds of relapse compared to placebo (OR ∼0.2 [0.09–0.53], *p* < 0.001), with the number of relapses being 8 out of 80 in the treatment group compared to 25 out of 78 in the placebo group (Achiron et al. 2015). It is important to highlight that from this trial was the use of alfacalcidol, which is an active analogue of vitamin D supplementation compared to the majority of studies using cholecalciferol, as alfacalcidol bypasses the hydroxylation steps that standard vitamin D supplementation undergoes. Figure 2 represents a visual summary of the directional effect estimated across key trials.

### 3.5 MRI Outcomes

The MRI data showed a contrasting association when compared to the relapse data, and that difference is actually one of the more interesting findings of this review. While most of the relapses showed to be statistically insignificant, a few trials showed significant reductions in inflammatory lesion activity on MRI even in the absence of a clinical relapse benefit.

The SOLAR trial found that vitamin D supplementation significantly reduced combined unique active lesions compared to placebo (IRR 0.68 [0.52–0.89], *p* = 0.0045) as well as a significant reduction in T2 lesion volume change (*p* = 0.035) (Hupperts et al. 2019). Soilu‐Hänninen et al. (2012) also found a significant reduction in GELs when comparing vitamin D supplementation versus placebo groups (0.1 vs. 0.6, *p* = 0.004), though within the same trial, there was no significance found in the T2 lesion volume trend (*p* ≈ 0.105). In the post hoc analysis of the FREEDOMS fingolimod trials by Hongell et al. (2018), there was a higher proportion of T2 lesion‐free scans at both 12 months (*p* = 0.038) and 24 months (*p* = 0.009), while also showing an improved percent brain volume change at 12 months (*p* = 0.018), although that same benefit was not sustained by 24 months (NS). Galus et al. (2025) found a significant reduction in new T2 lesions at 24 months in the supplemented group compared to non‐supplemented (*p* = 0.034), though GELs at the same timepoint did not reach significance (*p* = 0.094). Taken together, these findings were inconsistent and not reproducible across studies.

Importantly, a number of trials found no meaningful MRI benefit. The VIDAMS trial reported no significant change in new or enlarging T2‐weighted lesions (rate ratio 1.47 [0.88–2.46], *p* = 0.14), brain parenchymal volume change (−0.81 vs. −0.29, *p* = 0.13), or normalized gray matter volume change (−0.87 vs. −0.15, *p* = 0.08) at 2 years (Cassard et al. 2023). Mosayebi et al. (2011) reported no difference between control and treatment groups in the mean number of GELs during their 6‐month treatment period. The CHOLINE trial showed mixed results with some T1 lesion measures nominally favoring vitamin D, but new and enlarging T2‐weighted lesions and GELs showed *p* > 0.05 (Camu et al. 2019). Table 5 summarizes MRI outcomes and significance for included studies.

**TABLE 5:** MRI outcomes across included primary studies.

Study (year)	Design	*N*	MRI outcome	Effect (Vit D vs. control)	*p*‐value	Significant
Hupperts (2019)	RCT (add‐on IFNβ‐1a)	229	CUA lesions (new Gd+ T1, new T2, or enlarging T2)	IRR 0.680 (0.520–0.890)	0.0045	Yes
Hupperts (2019)	RCT (add‐on IFNβ‐1a)	229	T2 lesion volume change	Difference in mean ranks ∼−0.074	0.035	Yes
Soilu‐Hänninen et al. 2012)	RCT (add‐on IFNβ‐1b)	66	Gd+ lesions	0.100 vs. 0.600	0.004	Yes
Soilu‐Hänninen et al. 2012)	RCT (add‐on IFNβ‐1b)	66	T2 lesion volume	Smaller increase (Vit D)	0.105	No
Hongell et al. (2018)	Post hoc (FREEDOMS/FREEDOMS II)	829	Free of new/enlarging T2 lesions	Favored daily Vit D users at M12 and M24	0.038 (M12); 0.009 (M24)	Yes
Hongell et al. (2018)	Post hoc (FREEDOMS/FREEDOMS II)	829	Percent brain volume change (PBVC)	Better in Vit D users at M12; NS at M24	0.018 (M12); NS (M24)	Partial
Camu (2019)	RCT (add‐on IFNβ‐1a)	129^a^	New T1 lesions/T1 hypointense volume	Nominally favored Vit D	NS (select measures)	No
Camu (2019)	RCT (add‐on IFNβ‐1a)	129^a^	New/enlarging T2 lesions; Gd+ lesions	No significant difference	NS	No
Mosayebi et al. (2011)	RCT	62	Gd+ lesions	No significant difference reported	NS	No
Cassard (2023)	RCT (add‐on GA)	172	New/enlarging T2 lesions (2y)	Rate ratio 1.470 (0.880–2.460)	0.140	No
Cassard (2023)	RCT (add‐on GA)	172	Brain parenchymal volume change (2y)	−0.810 vs. −0.290	0.130	No
Cassard (2023)	RCT (add‐on GA)	172	Normalized gray matter volume change (2y)	−0.870 vs. −0.150	0.080	No
Galus et al. (2023) (observational)	Prospective cohort	133	New T2 lesions (24 mo)	0.400 vs. 0.800	0.034	Yes
Galus et al. (2023) (observational)	Prospective cohort	133	Gd+ lesions (24 mo)	0.000 vs. 0.800	0.094	No

*Note*: CUA = combined unique active; GA = glatiramer acetate; Gd+ = gadolinium‐enhancing; IFNβ = interferon beta; IRR = incidence rate ratio; M12 = month 12; M24 = month 24; mo = months; MRI = magnetic resonance imaging; NS = not significant; PBVC = percent brain volume change; RCT = randomized controlled trial; T2 = T2‐weighted; Vit D = vitamin D; 2y = 2 years. Significant column: Yes = *p* < 0.05 favoring vitamin D; No = *p* ≥ 0.05; Partial = significant at one timepoint only.

^a^ CHOLINE enrolled 129 participants; approximately 90 completed the full 96‐week follow‐up.

This trend is suggestive of showing that if vitamin D supplementation has any effect in MS, it may be more detectable at the radiographic level than at the level of clinical relapses. It is important to indicate the distinction between MRI lesion suppression and fewer relapses and take note that the two are not the same thing, and evidence here shows they do not always track together. Whether that reflects a real but clinically incomplete effect, or whether it is noise driven by small sample sizes and variability between trial designs, is something that current evidence cannot definitively answer. It is also possible that any immunomodulatory effect of vitamin D supplementation is real but modest in immensity, detectable on radiographic imaging but insufficient to meaningfully alter relapse frequency.

### 3.6 Reporting Bias

The potential for reporting bias cannot be excluded in this review. Several included trials were small and underpowered for relapse outcomes. Additionally, at least two studies did not report MRI data despite specifying imaging as a prespecified outcome, potentially underrepresenting statistically insignificant MRI findings in the published literature (Burton et al. 2010; Kampman et al. 2012).

### 3.7 Certainty of Evidence

No formal GRADE assessment was performed, and interpretation is qualitative.

## 4 Discussion

This review evaluated whether vitamin D supplementation reduces relapse frequency and MRI activity in adults with MS. Three findings emerge from the evidence: vitamin D supplementation does not consistently reduce relapse frequency, a group of trials suggest a possible radiographic signal at MRI level, and clinical relevance remains uncertain. The relapse findings were largely insignificant, which is consistent with results of previous systematic reviews and meta‐analyses as shown in Table 6. For example, the Cochrane review by Jagannath et al. (2018) found no consistent ARR benefit across 12 trials. Similar to this review, Jagannath et al. also incorporated both RCT and non‐randomized evidence and rated the overall certainty of findings as low. Additionally, pooled analyses by Mahler et al. (2024) and Serag et al. (2025) also reported inconsistent or insignificant relapse effects, reinforcing the consistency of inconclusive relapse outcomes across independent reviews. Not all prior reviews reached the same conclusion regarding relapse. Hanaei et al. (2021) reported an overall relapse reduction, a finding that diverges from the null relapse result of the present review. These discrepancies may reflect differences in included study populations, vitamin D dosing regimens, or outcome definitions across reviews, which limit direct comparability.

**TABLE 6:** Summary of previous systematic reviews and meta‐analyses.

Study (year)	Evidence type	Included studies/participants	Main relapse conclusion	Main MRI conclusion
Jagannath et al. (2018)	Cochrane SR/MA	12 trials; ∼933 participants	No consistent ARR benefit	Inconsistent MRI benefit; low certainty
Zheng et al. (2018)	SR/MA	6 RCTs; *n *≈ 337	ARR pooled effect small/inconsistent	MRI outcomes NR/varied
Hanaei et al. (2021)	SR/MA	*n *≈ 401	Reported relapse reduction overall	MRI not central
Pozuelo‐Moyano et al. (2013)	SR (RCTs only)	Small set of RCTs	Mostly statistically insignificant across early trials	One positive trial; heterogeneity noted
Mahler et al. (2024)	SR/MA (add‐on D3)	9 RCTs; *n *≈ 867	Inconsistent relapse effects	Inconsistent MRI effects
Gill et al. (2024)	SR	16 studies	Majority of RCTs NS for relapse	MRI improvement reported in subset
Serag et al. (2025)	SR/MA	*n *≈ 1903 (varies by outcome set)	Overall ARR often NS; subgroup signals	New T2 lesions modestly reduced (reported MD)

*Note*: MA = meta‐analysis; MD = mean difference; NR = not reported; NS = not significant; RCT = randomized controlled trial; SR = systematic review; T2 = T2‐weighted.

The MRI findings across the studies were more nuanced. Several trials showed a reduction in inflammatory lesions despite statistically insignificant relapse outcomes, with six reporting statistically significant changes. This suggests that if vitamin D supplementation has any effect in MS, it may be more detectable through radiographic testing than clinical outcomes (Hupperts et al. 2019; Soilu‐Hänninen et al. 2012). This discrepancy may reflect the relative sensitivity of MRI to subclinical inflammatory activity, which can occur without producing a clinically apparent relapse. Lesion formation and resolution are continuous processes that MRI can capture quantitatively, whereas relapse is a binary clinical event that may not register smaller reductions in disease activity. Whether vitamin D represents a real immunomodulatory effect or noise pertaining to trials is something that current evidence cannot definitively answer. This pattern is echoed in the secondary literature summarized in Table 6: Zheng et al. (2018) and Mahler et al. (2024) similarly reported inconsistent MRI effects, while Serag et al. (2025) and Gill et al. (2024) reported modest reductions in new lesion activity, and Pozuelo‐Moyano et al. (2013) identified a single trial with a significant MRI finding despite an otherwise null relapse result. Taken together, both the primary and secondary evidence point toward MRI as a more sensitive, though still inconsistent, marker of disease activity than clinical relapse.

### 4.1 Review Strengths

This review has several methodological strengths. Study selection and full text screening were conducted independently by multiple reviewers, with discrepancies resolved by consensus, minimizing selection bias. Risk of bias was assessed using two validated tools appropriate to study design (RoB‐2 for randomized trials and ROBINS‐I for non‐randomized studies), which allowed for a rigorous evaluation of study quality. By synthesizing both relapse and MRI outcomes, this review also provided a broader evaluation of disease activity, than focusing on a single outcomes domain, allowing for identification of discrepancies between radiographic and clinical measures of disease activity.

### 4.2 Evidence Limitations

Several limitations reduce confidence in the findings of this review. Most of the included trials were generally small to moderate in size (ranging from 49 to 229 participants among primary RCTs) and varied in duration (12 weeks to 48 months). Vitamin D formulations, doses, and dosing intervals showed significant variation across trials, ranging from 1 µg/day of alfacalcidol up to 40,000 IU/day of cholecalciferol. This makes direct comparisons difficult and prevents a meaningful pooling of results. Baseline serum 25(OH)D levels were also varied by trial, with some being inconsistently reported, if data were given at all, so whether deficient patients responded differently is something that would require further investigation. Risk of bias was common in light of single‐center designs (Achiron et al. 2015; Mosayebi et al. 2011), inadequate blinding (Burton et al. 2010), and funding from pharmaceutical companies in a few studies (Achiron et al. 2015; Hongell et al. 2018; Camu et al. 2019). High clinical variability in study design, and patient population and inconsistent reporting of secondary outcomes (e.g., fatigue and quality of life) further reduced certainty.

### 4.3 Review Limitations

This review was not prospectively registered onto a public registry, though the PICO protocol was developed prior to the search and defined the primary outcomes before data collection began. The review restricted inclusion to English‐language publications, which may have missed relevant studies published in other languages. No meta‐analysis was performed due to heterogeneity in study designs, interventions, and reported outcomes; therefore, a systematic approach was used.

### 4.4 Implications

The evidence reviewed here would not support vitamin D supplementation as a standalone treatment or replacement of disease‐modifying therapy in MS. Vitamin D remains a reasonable supportive adjunct as it is inexpensive, widely available, generally well tolerated, and given that MS patients disproportionately have lower serum vitamin D levels. Correction of vitamin D deficiency is already addressed in existing clinical guidance, and the evidence presented here does not extend beyond that. High‐dose supplementation for disease modification is not supported by current data, and the evidence here does not provide a basis for expanding its clinical role.

Future research should prioritize larger multicenter RCTs that have adequate power for relapse as a primary endpoint, with standardized dosing protocols and baseline 25(OH)D serum levels being reported to improve comparison across studies. A longer follow‐up of at least 2–3 years is needed to capture differences in MRI activity and relapse accumulation to help clarify the potential of vitamin D supplementation beyond use as a correction of a deficiency in MS.

## Author Contributions

**Kamren Hall**: conceptualization, methodology, investigation, data curation, writing – original draft, writing – review and editing. **Jessica Renteria**: conceptualization, investigation, data curation, writing – original draft, writing – review and editing. **Elizabeth Fernandez**: conceptualization, investigation, data curation, writing – original draft, writing – review and editing. **Janique Abrahams**: conceptualization, investigation, data curation, writing – original draft, writing – review and editing. **Saajan Bhakta**: conceptualization, supervision, writing – review and editing.

## Funding

The authors have nothing to report.

## Ethics Statement

This project was exempt from IRB approval on account of being a systematic review.

## Conflicts of Interest

The authors declare no conflicts of interest.

## Other Information

A written PICO protocol was developed by the research team prior to initiation of the search, defining the population, intervention, comparator, and outcomes of interest. This review was not registered in a public registry, such as PROSPERO, and was not publicly deposited. This review received no financial support. The authors declare no competing interests. All extracted data are presented within the manuscript tables and are not separately available outside this publication.

## Data Availability

All data supporting the findings of this review are contained within the manuscript tables, no data are separately available outside this publication.
